# Edge effects in calling variants from targeted amplicon sequencing

**DOI:** 10.1186/1471-2164-15-1073

**Published:** 2014-12-05

**Authors:** Ravi Vijaya Satya, John DiCarlo

**Affiliations:** Research and Foundation Department, QIAGEN Sciences, Inc, Frederick, MD USA

## Abstract

**Background:**

Analysis of targeted amplicon sequencing data presents some unique challenges in comparison to the analysis of random fragment sequencing data. Whereas reads from randomly fragmented DNA have arbitrary start positions, the reads from amplicon sequencing have fixed start positions that coincide with the amplicon boundaries. As a result, any variants near the amplicon boundaries can cause misalignments of multiple reads that can ultimately lead to false-positive or false-negative variant calls.

**Results:**

We show that amplicon boundaries are variant calling blind spots where the variant calls are highly inaccurate. We propose that an effective strategy to avoid these blind spots is to incorporate the primer bases in obtaining read alignments and post-processing of the alignments, thereby effectively moving these blind spots into the primer binding regions (which are not used for variant calling). Targeted sequencing data analysis pipelines can provide better variant calling accuracy when primer bases are retained and sequenced.

**Conclusions:**

Read bases beyond the variant site are necessary for analysis of amplicon sequencing data. Enzymatic primer digestion, if used in the target enrichment process, should leave at least a few primer bases to ensure that these bases are available during data analysis. The primer bases should only be removed immediately before the variant calling step to ensure that the variants can be called irrespective of where they occur within the amplicon insert region.

**Electronic supplementary material:**

The online version of this article (doi:10.1186/1471-2164-15-1073) contains supplementary material, which is available to authorized users.

## Background

Advances in high-throughput sequencing have enabled the adoption of sequencing for various applications in research and clinical diagnostics. In addition to lower per-base sequencing costs, one of the crucial factors in reducing per-sample sequencing costs is the ability to focus sequencing throughput on specific target regions of interest. Multiple enrichment strategies are currently in use for target enrichment [1]. These target enrichment strategies can be broadly classified into PCR-based methods and hybridization-based methods. Hybridization-based enrichment is by far the most widely used approach for large target regions such as targeted exome sequencing [2], in which all protein-coding regions and untranslated regions flanking them are targeted. Both hybridization-based and PCR-based enrichment strategies are often used for smaller target regions.

**Figure 1:**
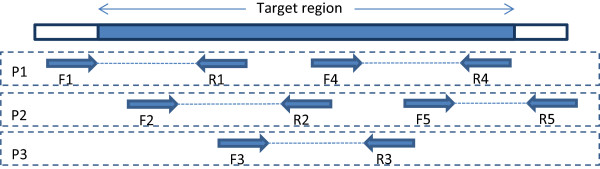
**Multiplex PCR based target enrichment.** A typical design used for targeted amplicon sequencing. The target is tiled with overlapping amplicons. Each amplicon is represented by a forward primer (F#) and a reverse primer (R#). Any two amplicons that overlap with each other are added to separate multiplex PCR pools to avoid undesired PCR products. The design above requires three multiplex PCR pools denoted by P1, P2 and P3.

PCR-based target enrichment, or targeted amplicon sequencing, is accomplished either by a few high dimension multiplex PCR reactions (AmpliSeq™ from Life Technologies, GeneRead from QIAGEN), or using thousands of single-plex PCR reactions accomplished using microfluidics [3]. Targeted amplicon sequencing offers some distinct advantages over hybridization-based methods, which include faster reaction times and the ability to start with smaller amounts of input DNA. A typical amplicon design for targeted sequencing consists of small overlapping amplicons that tile the target region as shown Figure 1. Ideally, the length of these amplicons is not much larger than the average read length, to ensure that most bases in the amplicon insert are covered by reads in either direction. At any single position, having the reads from both the strands enables the identification of strand-specific sequencing artifacts. The products of PCR enrichment include the primers on both ends. However, these primers are not native to the sample, and need to be removed before variant calling so that they do not distort the variant calls from other amplicons that overlap these primers. This primer removal can be accomplished before sequencing through enzymatic primer digestion [4], or after sequencing, by trimming the primer bases from the reads. Enzymatic primer digestion before sequencing makes better utilization of sequencing throughput. Because the primers are removed before sequencing, the sequencing capacity is not wasted in sequencing the primers. As a result, the amplicon design can accommodate longer amplicons than otherwise possible, which means that the target region can be covered with fewer amplicons, ultimately resulting in higher overall read depth per amplicon.

**Figure 2:**
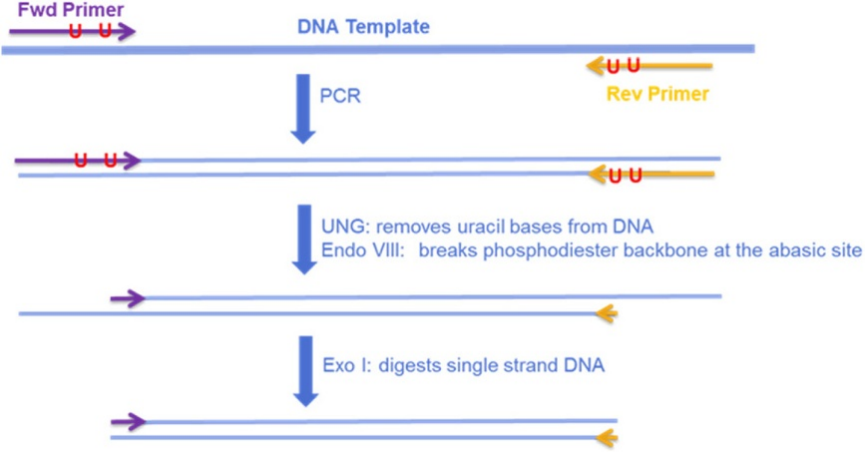
**Enzymatic primer digestion procedure.** The ‘*U*’s indicate Uracil bases in the primers. These bases are placed as close the 3’ end of the primer as possible to maximize the number of primer bases removed.

Enzymatic primer digestion is generally accomplished by substituting one or two Thymine (*T*) bases in the primer with Uracil (*U*) bases [4]. During the PCR reaction, Adenine (*A*) bases are incorporated in positions complimentary to the *U* bases in the primer. The double-stranded DNA product after PCR is treated with uracil-N-glycosylase to remove the primer strand until the furthest 3’ *U* base. This results in single-stranded overhangs at both ends of double-stranded PCR product, as shown in Figure 2. The single-stranded overhangs are removed using exonuclease. A *U* base is generally placed as close to the 3’ end of the primer as possible, in order to maximize the number of primer bases digested and minimize the number of primer bases that are sequenced.

**Figure 3:**
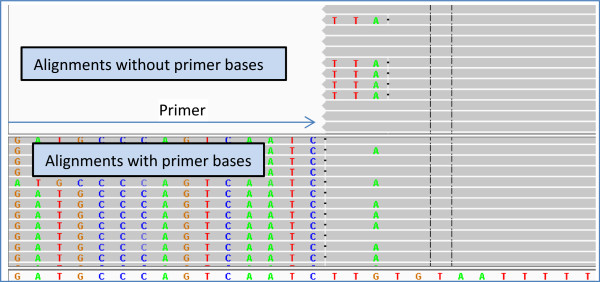
**Comparison of read alignments with and without primer bases.** The top panel shows alignments of reads without primer bases and the bottom panel shows alignments of reads with primer bases. For reads without the primer bases, the *G*- > *A* variant near the end of the read causes the read aligner to exclude a portion of the amplicon insert from the alignment, while the reads without the variant are aligned completely. Because the variant base is not part of the alignment, it is impossible to call the variant. In contrast, for reads with the primer bases (bottom panel), the complete amplicon insert region is aligned, thereby allowing the *G*- > *A* variant to be called. In the bottom panel, the primer bases have been soft-clipped by us after obtaining the alignments with the complete reads; *i.e.*, the read aligner did not soft-clip the alignments in the bottom panel.

However, enzymatic primer digestion also has a downside. Alignments of sequencing reads to a reference can be inaccurate towards the ends of a read. This is especially true when there is a variant near an edge of the read. The mismatch due to the variant might cause all the bases from the variant to the edge of the read to be excluded from the alignment, otherwise known as ‘soft-clipping’ of the read bases. An example of this scenario is shown in the top half of Figure 3 where the alignments result in a false-negative variant call. In some cases, a variant near the end of a read can cause mis-alignments that can lead to a false-positive variant call in addition to a false-negative. An example of this scenario would be a deletion near the end of a read. The aligner might prefer an alignment without the deletion near the end, which can lead to a false-positive SNP call in addition to missing the deletion. An example of this scenario is shown in Additional file 1: Figure S1. The effect of these inaccurate alignments would be negligible when random fragments are sequenced, because the read starts and read ends are all staggered after random fragmentation. Any individual misalignment will not impact the overall variant calls as long as the read depth is greater than a few reads. However, the same is not true when PCR products are sequenced. All the 5’ ends of reads from amplicon sequencing start at the same position, and the 3’ ends also tend to cluster around a single position. This means that any misalignment towards the edges of read can be pervasive throughout the read stack. In this paper, we show that these misalignments reduce the likelihood that a variant near the edge of an amplicon is called.

We present results from variant calling on simulated amplicon sequencing reads where each read has a variant near the edges of the amplicon, with varying number of primer bases remaining after primer digestion. Our analysis suggests that primer bases should be part of read alignments and subsequent post-processing of the alignments to ensure variant calling with high sensitivity. If enzymatic primer digestion is used, we argue that at least a few primer bases should be left undigested.

## Methods

We conducted a simulation study to empirically evaluate the effect of distance from the edge of read on the ability to call a variant. The purpose of this study is to create simulated amplicon sequencing reads with variants at the ends of each amplicon insert and primer digestion at varying distances from the amplicon insert, and then observe how many of these variants can be recovered through a standard analysis pipeline. For simplicity in presentation, we simulated only point mutations at the amplicon ends.

### Amplicon design

To ensure that the primer sequences and insert sequences are realistic representations of actual target enrichment, we targeted coding regions of 167 genes that are of high interest in studying various cancers. The amplicon design consisted of 8,035 primer pairs that cover 94.5% of the protein coding regions in these 167 genes. These 8,035 primer pairs were divided into four pools, with roughly the same number of primer pairs in each pool. The lengths of the complete amplicons ranged from 100 to 180 bp, and the amplicon inserts ranged from 53 to 146 bp. The primer lengths varied from 17 to 28 bp.

### Simulated reads

**Figure 4:**
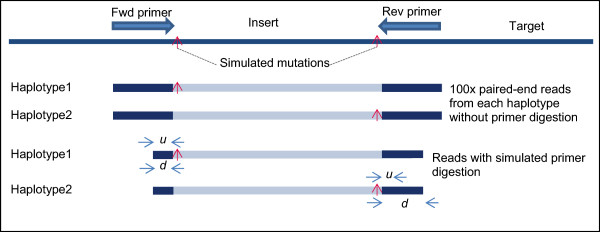
**Simulated reads.** Simulated reads are generated with mutations at each end of the amplicon insert. The minimum distance between the Uracil base in the primer and the 3’end of the primer is indicated by *u*. Only Thymine bases in the primer can be replaced by a Uracil base for primer digestion, so the actual distance from the Uracil base to the 3’ end of the primer (indicated by *d*) is dependent on the primer sequence, and can be much greater than *u*. The figure shows a scenario where *d* is the same as *u* on one end of the amplicon, but *d* is much greater than *u* on the other end of the amplicon.

For each amplicon, we generated a point mutation at the first base after the primer by mutating the base to one of the three non-reference bases at that position. In situations where the 5’ end of an amplicon insert is at the same position as the 3’ end of some other amplicon insert (*i.e.*, instances where two adjacent amplicon inserts overlap by a single base), we generated the mutation for only one of the amplicons to ensure that we have no more than one point mutation at any reference position in the simulated data. In total, we generated 15,871 point mutations. We took the complete sequence of each amplicon and created two haplotype sequences from each amplicon by incorporating exactly one mutation into each haplotype, as illustrated in Figure 4.

Next, we generated simulated paired-end Illumina reads at 100x read-depth from each haplotype using a modified version of the ART read simulator [5] that generates simulated reads from amplicon sequencing. In the primer design, we have anywhere between one to three amplicons overlapping any base in the target region. Hence, the actual allele frequency of the simulated variants was 50%, 25% or 16.67%, based on whether 1, 2, or 3 amplicons, respectively, covered that position in the target. We simulated primer digestion on these reads by trimming primer bases up to the first Thymine base that is at least *u* bases away from the 3’ end of the primer, for variable values of *u* (*u* ∈ {1,2,3,4,5,6,7,8}). The base at the 3’ end of the primer (corresponding to *u* = 0) is never replaced by ‘*U*’, so we did not perform simulated primer digestion for *u* = 0.

### Data analysis

We analyzed the simulated reads with a standard pipeline that involves read alignment with BWA [6, 7], post-processing of the alignments with GATK indel realigner, GATK base quality score recalibrator (BQSR), and GATK base alignment quality (BAQ) computation [8], trimming of the residual primer bases using custom scripts, and variant calling with GATK Unified Genotyper [9]. We analyzed the resulting variant calls to study the effect of primer digestion on recoverability of the simulated variants. The parameters used for various steps in the analysis and versions of software used are provided in Additional file 1: Supplementary Methods.

## Results

The results from variant calling on simulated data clearly show decreased sensitivity for smaller values of *u*. A smaller value of *u* allows most of the primer sequence to be digested, effectively bringing the variant near the end of the amplicon insert into the variant calling blind spot near the end of the read. This mostly leads to false-negatives in the variant calling results, though it is also possible to see some false positives due to misalignments near the read ends.

### Effect of primer digestion

**Figure 5:**
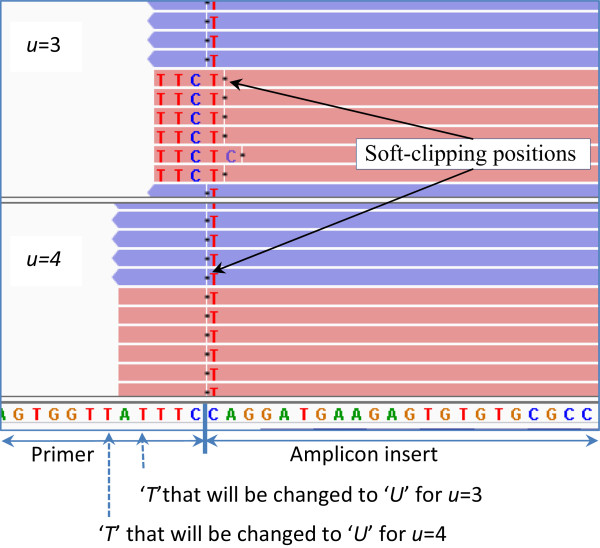
**An instance of misalignment at** ***u*** **= 3 which is corrected for larger values of** ***u*** **.** The top panel shows simulated reads with *u* = 3, and the bottom panel shows simulated reads with *u* = 4. The reads in the forward direction are in pink and the reads in the reverse direction are in blue. A *C*- > *T* mutation (highlighted) has been inserted into some of the reads at the first position in the insert. When *u* = 3, only three bases of the primer are left in the read after primer digestion. The *C*- > *T* mismatch causes the alignments of all the forward reads to be clipped to exclude the variant from the alignment. Breaks in the reads indicate soft-clipping positions. At *u* = 4, five bases of the primer are left, which was sufficient to avoid the misalignments.

The sensitivity of variant calling for different values of *u* is shown in Table 1*.* Only 82.17% of the simulated variants could be recovered (called from the simulated reads) for *u* = 1, *i.e.*, when the minimum distance of the simulated Uracil base from the 3’ end of the primer is 1. Only 97.5% of the simulated variants were recoverable at *u* = 2. The sensitivity does not reach 100% until *u* = 8. There was one false positive insertion called at *u* =1. No false positives occurred in data sets with *u* > 1. Because the simulated reads are generated so that there is ample read depth to call each of the simulated variants, this clearly shows that the variant calls are less accurate near the read ends. An example of misalignment at *u* = 3 which led to a false-negative is presented in Figure 5.

**Table 1:** **Sensitivity of calling simulated variants for different values of** ***u***

***u***(minimum distance of the Uracil base from 3’ end of primer)	Median value of ***d***(actual distance of the Uracil base from the 3’ end of the primer)	No. of simulated variants	Recovered variants	% of variants recovered	No. of false-negatives
1	2	15,871	13,041	82.17	2,830
2	3	15,871	15,474	97.50	397
3	5	15,871	15,834	99.77	37
4	6	15,871	15,858	99.92	13
5	7	15,871	15,866	99.97	5
6	8	15,871	15,869	99.99	2
7	9	15,871	15,870	99.99	1
8	10	15,871	15,871	100.00	0

### Extent of variant calling blind spots

The results in Table 1 are based on minimum distance of the simulated Uracil base from the 3’ end of the primer. The actual position of the simulated Uracil base depends on the primer sequence and the position of the first Thymine base beyond the minimum distance within primer sequence. Therefore, for any value of *u*, the actual distance between the variant (which is at the end of the amplicon insert, as shown in Figure 4) and the simulated Uracil base (denoted by *d*) can be much greater than *u*. To study how far the blind spots near the read ends extend, we examined the sensitivity of variant calling for different values of *d*. Analysis of variation of sensitivity for different values of *d* will help us identify the actual extent and severity of the blind spots near the read ends.

To study the extent of these blind spots, we combined the simulation results from all the different values of *u*, and tabulated the recoverability based on *d* irrespective of the actual value of *u* used for the simulated primer digestion. These results are shown in Table 2. Based on these results, only 66% of the simulated variants were recoverable when the variant is only 1 bp away from the end of the read. Less than 93% of the simulated variants were recoverable when the variant is 2 bp away from the end of the read. More than 99% of the variants were recoverable for all values of *d* ≥ 3. All simulated variants were recoverable for values of *d* ≥ 8. Small numbers of variants were non-recoverable for *d* between 4 and 7. Based on these results, we can see that the blind spots for point mutations can extend as far as 7 bases from the edges of a read.

**Table 2:** **Number of simulated variants and the recoverability of these variants for different values of** ***d***

***d***	No. of simulated variants	Recovered variants	% of variants recovered	No. of false-negatives
1	5,809	3,833	65.98	1,976
2	4,722	4,371	92.57	351
3	4,599	4,580	99.59	19
4	4,371	4,364	99.84	7
5	4,164	4,162	99.95	2
6	4,435	4,435	100.00	0
7	4,402	4,401	99.98	1
8	4,272	4,272	100.00	0
≥9	11,599	11,599	100.00	0

### Factors contributing to variant calling blind spots

Multiple factors affect the extent of the variant calling blind spots. The choice of the aligner and the parameters used in the alignment and post-processing steps will have an impact on the variant calls. In our default pipeline, we ran BWA with a large 5’-end soft-clipping penalty (-L 1000,5). This effectively discourages any soft-clipping at the 5’-end of the reads, thereby forcing the variant to be part of the alignment as long as the variant is not near the 3’end of the read. In our simulation, at least half of the reads with each variant have the variant near the 5’end of the read. As a result, most of the variants can be recovered from the alignments as long as the alignments are not subject to any further post-processing steps. This is evident from the results in Table 3, which shows that only 8 out of 15,871 variants were not recovered for *u* = 1 when both BAQ and BQSR steps are omitted. However, adding the BQSR step increases the false-negatives to 669 for *u* = 1. Adding the BAQ step has an even bigger impact, with 3,725 false-negatives for *u* = 1. This is to be expected, since any variant near the end of a read greatly reduces the confidence in the alignments, as alternate alignments without the variant become more plausible. A detailed analysis of how variants affect the BAQ scores is presented in Additional file 1: Supplementary Methods. Having the BQSR step before the BAQ step reduces the impact of the BAQ step: the false-negatives drop to 2,830 when both BQSR and BAQ steps are included. The exact mechanism of how the BQSR step affects the BAQ scores is not clear.

**Table 3:** **Number of false-negatives when different steps in the pipeline omitted**

***u***(minimum distance of the Uracil base from 3’ end of primer)	No. of simulated variants	False-negatives			
		Without BAQ and BQSR	With BQSR and without BAQ	With BAQ and without BQSR	Default pipeline (with both BAQ and BQSR)
1	15,871	8	669	3,725	2,830
2	15,871	9	23	775	397
3	15,871	3	3	136	37
4	15,871	1	1	59	13
5	15,871	0	0	35	5
6	15,871	0	0	20	2
7	15,871	0	0	23	1
8	15,871	0	0	28	0

## Discussion

Our results clearly show the presence of blind spots near the read ends in amplicon sequencing data. The actual extent and the root cause of these blind spots vary based on the aligner, the parameters used in the alignment and post-processing steps, and the types of the variants. Irrespective of the root cause of these blind spots, our results show that we can avoid problems in variant calling by simply including some primer bases in the read alignment (but subsequently excluding them for variant calling). The variants within the amplicon insert regions can be called with high accuracy as long as the blind spots in each read are confined to the primer bases.

For simplicity of illustration, we have only simulated point mutations in our analysis. Insertions and deletions (indels) near the read ends have a much bigger impact on the alignments, and hence might lead to even wider blind spots near the read ends. The actual impact of any indel will depend on the length of the indel, proximity to the edge of the read, and complexity of the nucleotide sequence around the indel. While it might not be possible to guarantee that an indel of any length near the end of a read can be correctly aligned, including as many primer bases as possible into the read will enable calling most variations near the amplicon ends.

In the simulations, we made the conscious decision to generate the variants on the amplicons rather than on the genome. If a variant is generated on the genome, it might be near the end of one amplicon, but in the middle of a second amplicon, due to the overlapping amplicons. In these situations, the variant might be called based on the reads from the second amplicon, but the observed allele frequency will be different from the actual allele frequency due to the misalignments in the first amplicon. The edge effects are much easier to isolate and study when the variant is present in only one amplicon.

Our results also cast doubt on the utility of enzymatic primer digestion. Given a fixed read length, enzymatic primer digestion enables the design of longer amplicons. Fewer amplicons will be necessary to cover the target region, which helps in reducing the complexity of the multiplex reaction. In addition, having fewer amplicons also helps in obtaining deeper coverage using the same sequencing throughput. However, the downside to these advantages is the significantly reduced ability to call variants near the ends of the amplicon.

A reasonable trade-off is to impose a minimum distance between the end of the amplicon insert and the first base from the 3’ end of the primer that can be removed through enzymatic primer digestion. Based on our simulation on a large number of genes, this distance must be at least 8 bases to make sure all possible point mutations at the end of the amplicon inserts are callable. However, this will significantly diminish the purported benefits of enzymatic primer digestion. Advances in sequencing technology and availability of longer read lengths might further reduce the need for enzymatic primer digestion.

## Conclusions

Design of targeted amplicon sequencing assays and analysis of the data from these assays requires awareness of the variant calling blind spots near the ends of a read. One approach to circumvent these blind spots is to ensure that at least a few bases of the primer are included at both ends of the read so that the variant calling blind spots are in effect moved into the primer binding regions, thereby allowing accurate variant calling within the amplicon insert region. To ensure maximum sensitivity, these primer bases should be intact during read alignment and post-processing steps and should be removed immediately before the variant calling step.

## Electronic supplementary material

Additional file 1: **Supplementary figures and supplementary methods.** (PDF 1 MB)
