# Supplementary material for: Edge effects in calling variants from targeted amplicon sequencing
Source: BMC Genomics. 2014 Dec 5;15(1):1073. doi: 10.1186/1471-2164-15-1073 (PMC4302139; doi:10.1186/1471-2164-15-1073)
Supplement: Supplementary file 1 — Additional file 1: Supplementary figures and supplementary methods. (PDF 1 MB) [file 12864_2014_6752_MOESM1_ESM.pdf]

Supplementary Materials

Figure S1

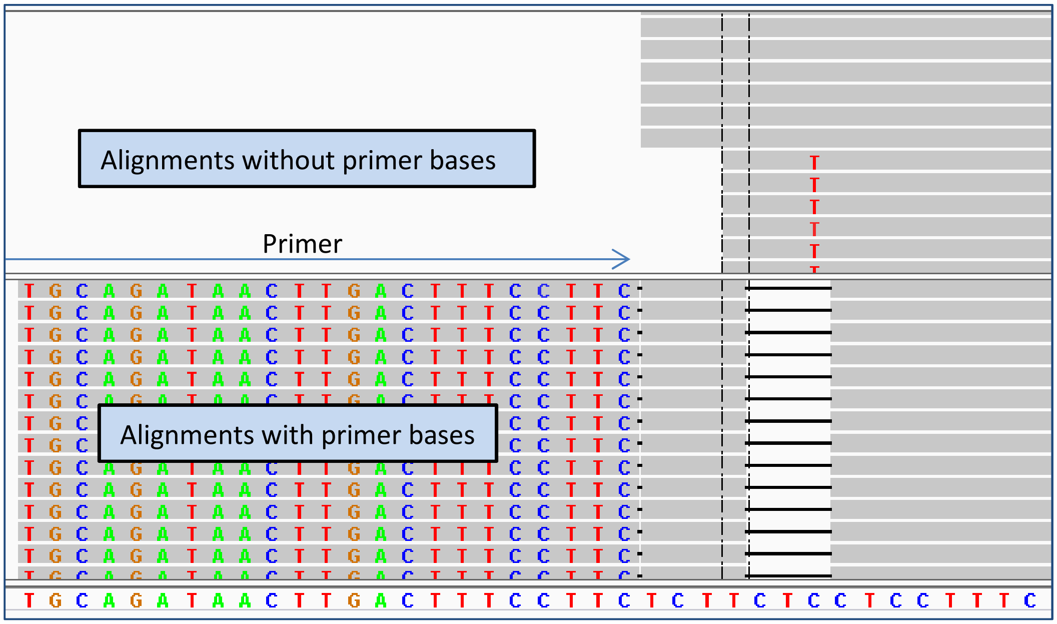

Figure S1: An instance where deletion near the end of the read leads to false-positive SNP call in addition to missing the deletion. As can be seen from the alignments with the primer bases (lower panel), there is a deletion of 'CTC' near the end of the insert. When the alignments are obtained without the primer bases (top panel), the aligner prefers to align these reads without the deletion, which leads to C->T false-positive variant call.

Supplementary Methods

Impact of variants on Base Alignment Quality (BAQ) computations

Results in Table 3 of the main text show that BAQ scores have the biggest impact on the variant calls. There could be two possible reasons for this: 1) a general lowering of the BAQ scores near ends of the read could be causing the false-negatives, or 2) the presence of the variant itself could be leading to a reduction in the BAQ scores. To study which one of these two factors has a greater impact, we compared the BAQ scores in reads with and without variants. Since we generate paired-end reads from each haplotype, and each haplotype has a single mutation (Figure 4 in the main text), one of the paired-end reads will have a variant near the 5' end, and while the other will not have any variants near the 5' end (we are not considering 3' ends of the reads in these comparisons due to the possibility of lower base qualities near the 3' end of the read). By comparing the BAQ scores in the reads with and without variants, we will be able to study the impact of the variants on the BAQ scores. A comparison of BAQ scores in these reads is presented in Figure S2 and Figure S3 for the u=1 and u=8, respectively.

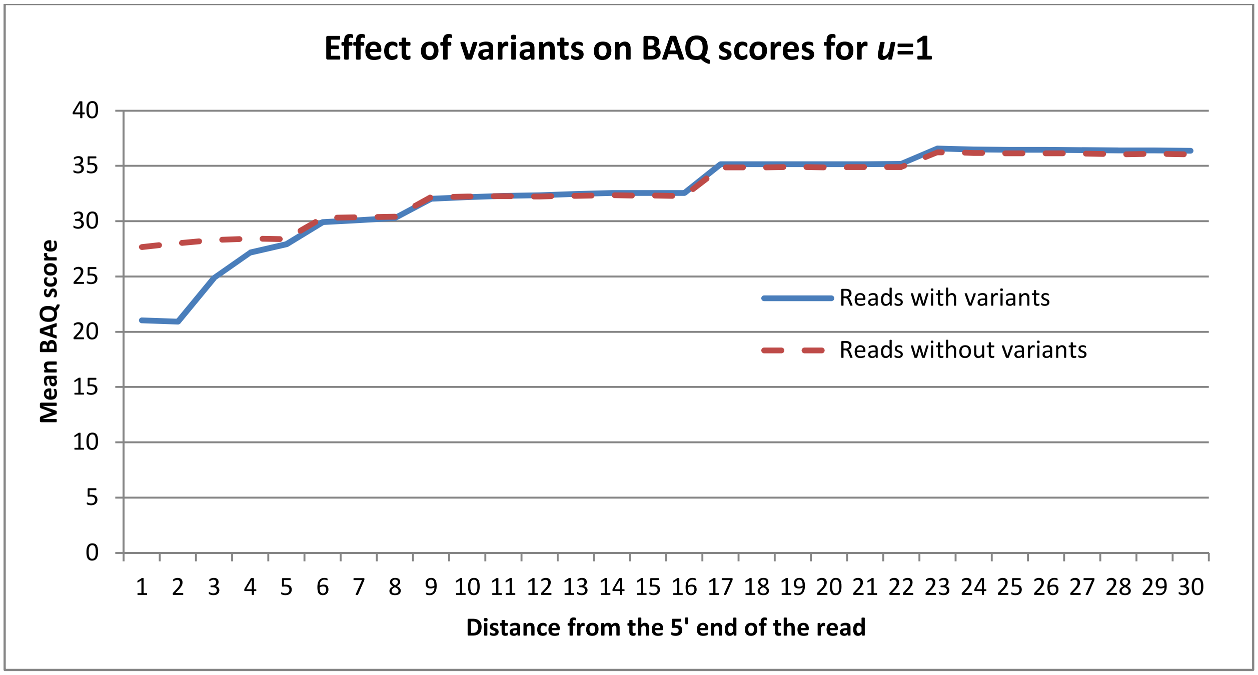

Figure S2: Comparison of BAQ scores in reads with and without variants for u=1. The BAQ scores are lower near the edges for all of the reads. Near the edges, the reads with the variants have significantly lower BAQ scores than reads without the variant. The BAQ scores of reads with and without the variants converge as we move away from the 5' end of the read.

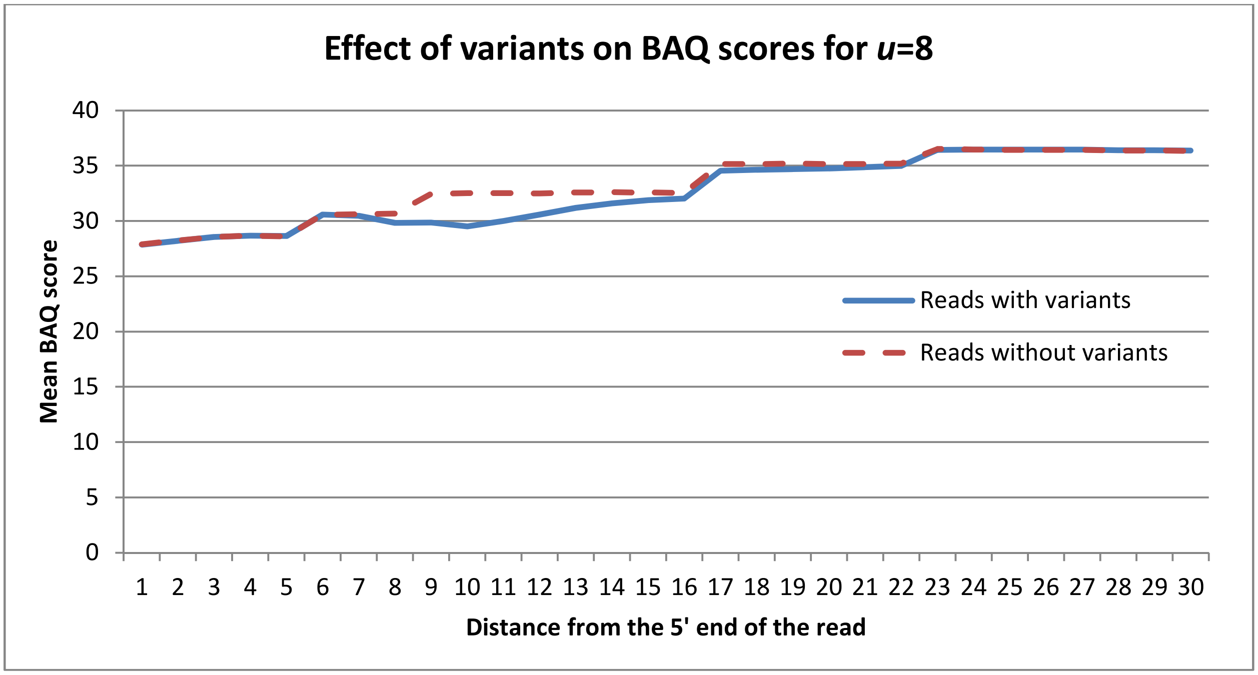

Figure S3: Comparison of BAQ scores in reads with and without variants for *u*=8. Again, all of the reads have lower BAQ scores near the edges. However, the difference in BAQ scores now moves away from the edges, as the variant is at least 8bp away from the edge. The BAQ scores are lower for reads with variants, but the difference is much smaller than the case when the variant is near the edge.

In general, the BAQ scores are lower near the ends of the read. However, the drop-off in BAQ scores is much steeper when the variant is close to the edge of the read (*u*=1, Figure S2). The drop-off in BAQ scores is much smaller when the variant is further away from the edge of the read (*u*=8, Figure S3). In both cases, the reads with variants have lower BAQ scores than reads without variants only in the vicinity of the variant. This clearly shows the presence of the variant causes a reduction in the BAQ scores, and this reduction is more significant near the edges of the read.

The reduction in BAQ score varies with actual distance of the variant from the edge of the read (*d*) and the sequence context near the variant. The histograms of BAQ scores at the variant position are shown in Figure S4, when the reads are generated with *u*=1. Some amplicons have a very low BAQ score at the variant base, while others have higher BAQ scores. Since we applied a minimum base quality threshold of 17 (the default in GATK Unified Genotyper), the variants with most of the reads below this threshold were not called. Reads without the variant have much higher BAQ score at the same positions (Figure S4-B). Splitting the reads based on *d*, we can see that most of the amplicons with low BAQ scores correspond to smaller values of *d* (Figures S4-C and S4-D).

The histograms of BAQ scores when the variant is much further away from the edge of the read (*u*=8) are shown in Figure S5. The BAQ scores are lower for reads with the variants, but the reduction is not large enough to cause false-negatives.

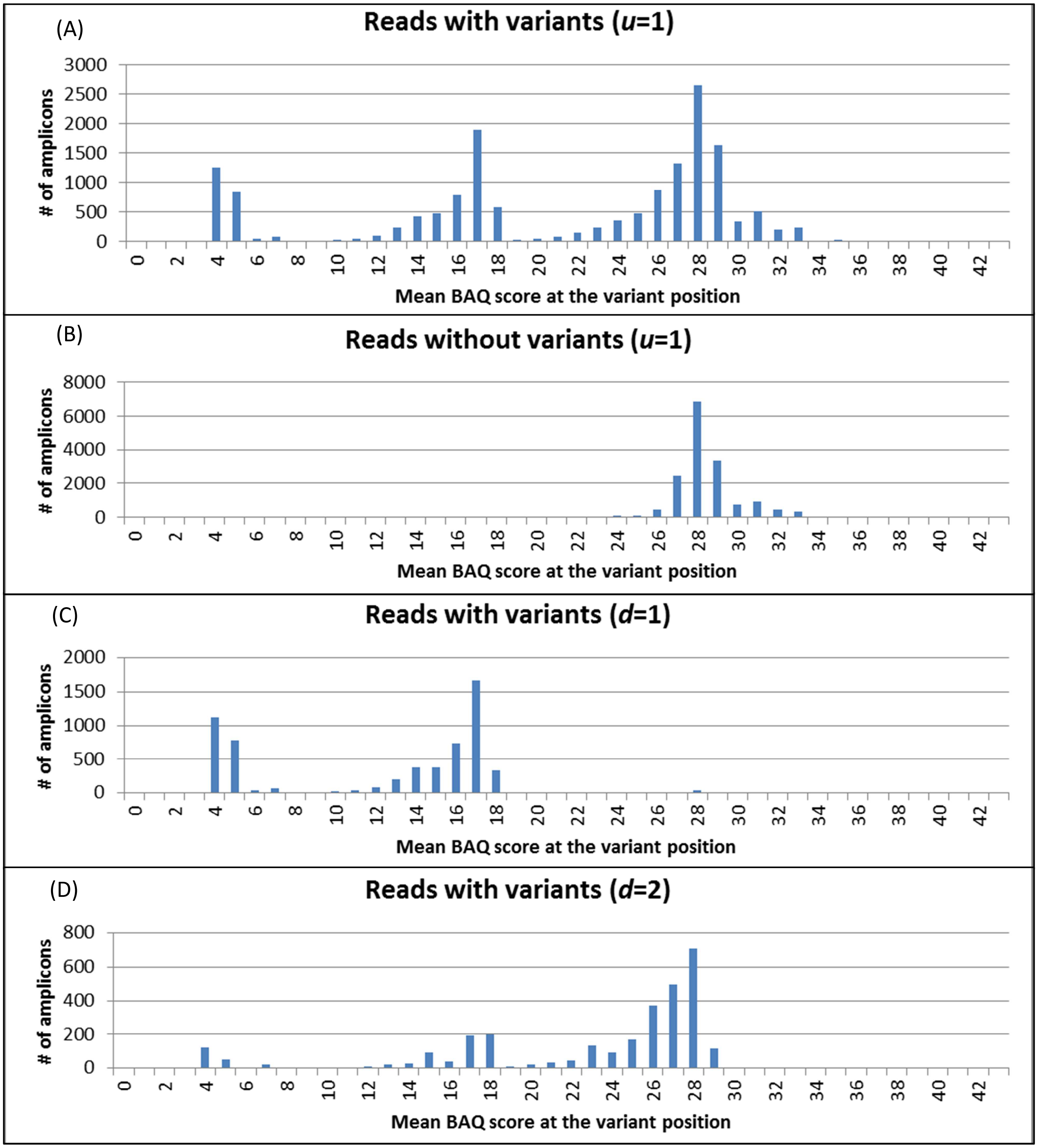

Figure S4: Histograms of mean BAQ scores at the variant position in reads from each amplicon for *u*=1. There are some amplicons with extremely low BAQ scores in reads with variants (A). However, the BAQ scores in the same amplicons are acceptable in reads without variants (B). Most of the low BAQ score amplicons correspond to smaller values of *d* (Figures C and D).

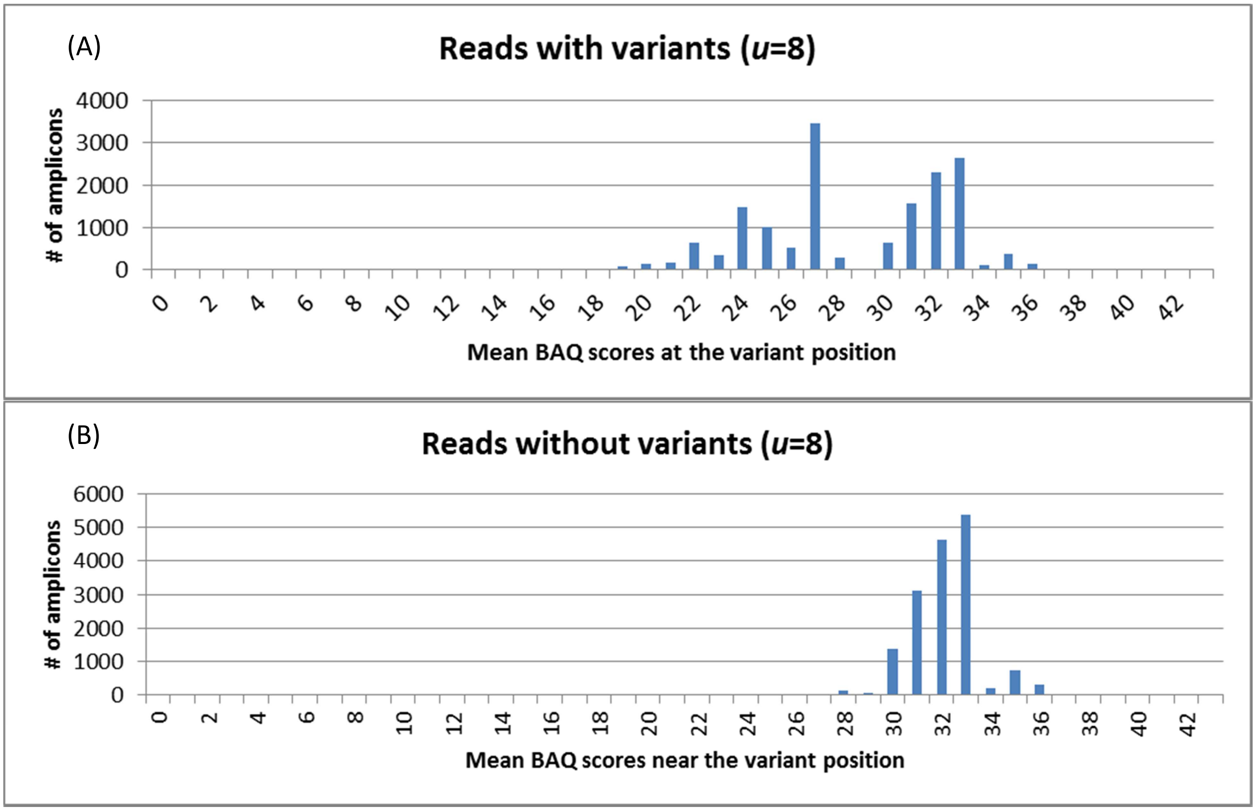

Figure S5: Histograms of mean BAQ scores at the variant position in reads from each amplicon for *u*=8. The reduction in BAQ score due to the variants is not significant enough to cause false-negatives.

Versions and command line arguments for various steps in the analysis pipeline are listed below:

Read generation: ART 1.5.1

art_illumina –i amplicons.fasta –o simreads_R –l 150 –f 200 –p –m 180 –s 10 –na -1 EmpMiSeq250R1.txt -2 EmpMiSeq250R2.txt

Alignment: BWA-MEM 0.7.5a-r422

bwa mem -M -L 1000,5 -t 8 ref.fasta simreads_R1.fq simreads_R2.fq | samtools view -Sb -F 256 -o reads01.bam –

Indel Realignment: GATK Indel Realigner (GATKLite 2.3-9)

java -XX:DefaultMaxRAMFraction=1 -XX:+UseParallelGC –jar GenomeAnalysisTKLite-2.3-9-gdcdccbb/GenomeAnalysisTKLite.jar -T RealignerTargetCreator -o realign.intervals -I reads01.bam --intervals ampliconInserts.bed -isr UNION --baq OFF -- --validation_strictness SILENT --interval_merging ALL -R ref.fasta -nt 8

java -XX:DefaultMaxRAMFraction=1 -XX:+UseParallelGC -jar GenomeAnalysisTKLite-2.3-9-gdcdccbb/GenomeAnalysisTKLite.jar -T IndelRealigner -I reads01.bam -o reads01.realigned.bam -LOD 5.0 --intervals ampliconInserts.bed -isr UNION --baq OFF --validation_strictness SILENT --interval_merging ALL –R ref.fasta -targetIntervals realign.intervals --disable_bam_indexing

Base Quality Score Recalibration: GATK Recalibrator (GATKLite 2.3-9)

Note: In running the BQSR step on the simulated reads, it is necessary to provide the simulated variants as known variants, as all the variants in the simulated data appear near the ends of the reads.

java -XX:DefaultMaxRAMFraction=1 -XX:+UseParallelGC -jar GenomeAnalysisTKLite-2.3-9-gdcdccbb/GenomeAnalysisTKLite.jar -T BaseRecalibrator -I reads01.realigned.bam -R ref.fasta --disable_indel_quals -knownSites simulated_variants.vcf -L primerPairs.bed -o reads01.recal.grp

java -XX:DefaultMaxRAMFraction=1 -XX:+UseParallelGC -jar GenomeAnalysisTKLite-2.3-9-gdcdccbb/GenomeAnalysisTKLite.jar -T PrintReads -I reads01.realigned.bam -R ref.fasta -BQSR reads01.recal.grp -o reads01.recal.bam -nct 8

Base Alignment Quality Computation: GATK PrintReads (GATKLite 2.3-9)

java -XX:DefaultMaxRAMFraction=1 -XX:+UseParallelGC -jar GenomeAnalysisTKLite-2.3-9-gdcdccbb/GenomeAnalysisTKLite.jar -T PrintReads -I reads01.recal.bam -R ref.fasta --baq RECALCULATE --baqGapOpenPenalty 30.0 -o reads01.baq.bam -nct 8

Primer Trimming

Custom python scripts using pysam library to trim away primer bases

Variant Calling: GATK Unified Genotyper (GATKLite 2.3-9)

java -XX:DefaultMaxRAMFraction=1 -XX:+UseParallelGC -jar GenomeAnalysisTKLite-2.3-9-gdcdccbb/GenomeAnalysisTKLite.jar -T UnifiedGenotyper -L ampliconInserts.bed -dcov 2500 –o all_variants.vcf -I reads.trimmed.bam --genotype_likelihoods_model BOTH -minIndelFrac 0.2 --min_base_quality_score 17 --standard_min_confidence_threshold_for_calling 30.0 --standard_min_confidence_threshold_for_emitting 30.0 --baq CALCULATE_AS_NECESSARY --baqGapOpenPenalty 30.0 --validation_strictness STRICT --interval_merging ALL -R ref.fasta -nt 8
